# Relationship between the sentiment of nursing notes and one-year mortality of elderly sepsis patients

**DOI:** 10.1371/journal.pone.0323686

**Published:** 2025-05-16

**Authors:** Li Sheng, Qin Zheng

**Affiliations:** Operating room, Quzhou People’s Hospital, Quzhou, Zhejiang, China; National Trauma Research Institute, AUSTRALIA

## Abstract

**Objectives:**

This study aimed to explore the correlation between the sentiment of nursing notes and the one-year mortality of sepsis patients.

**Methods:**

The box plot was used to compare the differences in sentiment polarity/sentiment subjectivity between different groups. Multivariate logistic regression was used to explore the correlation between sentiment polarity/sentiment subjectivity and one-year mortality of elderly sepsis patients. Ridge regression, XGBoost regression, and random forest were used to explore the importance of sentiment polarity and subjectivity in the one-year mortality of elderly sepsis patients. Restricted cubic spline (RCS) was used to explore whether there was a linear relationship between sentiment polarity, sentiment subjectivity and the one-year mortality of elderly sepsis patients. Kaplan-Meier (KM) curve was used to explore the relationship between the sentiment polarity (or sentiment subjectivity) and the 1-year death of the patient.

**Results:**

Compared with the control group, the one-year mortality group year had lower sentiment polarity and higher sentiment subjectivity. Sentiment polarity and sentiment subjectivity were independently related to the one-year mortality of elderly sepsis patients. There was a linear relationship between sentiment polarity and the one-year mortality of elderly sepsis patients. At the same time, there was a nonlinear relationship between sentiment subjectivity and the one-year mortality of elderly sepsis patients.KM.

**Conclusions:**

The sentiment of nursing notes was correlated with the one-year mortality of elderly sepsis patients.

## 1 Introduction

Sepsis is a serious life-threatening disease, which is characterized by the imbalance of immune response caused by infection and life-threatening multiple organ dysfunction. At present, about 48.9 million people in the world suffer from sepsis, and 11.1 million people die of sepsis [1]. Sepsis cases mainly occur in low-income or middle-income countries. The prevalence rates of severe sepsis in surgical Intensive Care Unit (ICU) and general ICU in China were 8.7% and 37.3% respectively [2]. The prevalence of sepsis in the ICU in India was 33.2% [3]. The overall prevalence of sepsis in ICU is 24.5% in low-middle-income areas in Asia [4]. The overall prognosis of sepsis patients is still poor despite the improvement of medical technology. A systematic review indicated that the mortality rate of sepsis patients in China was 29.0% [5] The in-hospital mortality rate of sepsis in the American population is 20.52% [6]. Therefore, it is necessary and urgent to actively explore the factors related to the prognosis of sepsis.

Sentiment analysis is a process of analyzing, processing, inducing, and reasoning subjective texts with sentiment color, which is mainly based on text data and is the main content of natural language processing. The purpose of sentiment analysis is to understand the author’s critical attitude (support or opposition to likes or dislikes, etc.) or sentiment state (happiness, anger, sadness, fear, etc.) towards an entity (including products, services, people, organizations, events, and topics) in the text. In recent years, sentiment analysis has been gradually extended to the medical field. It is found that keyword extraction and sentiment analysis based on discharge reports have certain predictive performance for 30-day unplanned readmission [7]. Sentiment in nursing notes was correlated with 30-day mortality and survival rate of ICU patients [8].

At present, there are few studies on sepsis and the sentiment of nursing notes. The existing studies mainly focus on the short-term prognosis of sepsis patients, but no research has focused on the long-term prognosis. Therefore, this study explored the correlation between the sentiment of nursing notes and the one-year mortality of sepsis patients.

## 2 Methods

### 2.1 Data source and indicators

The patient data came from the Medical Information Mart for Intensive Care (MIMIC)-IV database, which was an open database containing the data of all ICU patients in internal medicine and surgery at Beth Israel Deaconess Medical Center of Harvard Medical School [9].

### 2.2 Inclusion and exclusion criteria

Inclusion criteria: diagnosed with sepsis, older than 60 years of age.

Exclusion criteria: (1) Missing time of death after discharge from hospital, hospital death. (2) Miss clinical records. (3) Note writing fuzzy cannot be identified; (4) Have been identified as wrong notes by the doctor; (5) Notes less than 12 hours before death.

### 2.3 Sentiment analysis

Python and Text Blob were used to extract sentiment polarity and sentiment subjectivity from nursing records [8]. Given a string of texts, the Pattern module of TextBlob was marked, retrieved, and calculated. Specifically, the module includes a dictionary of English adverbs and adjectives, which can be mapped to the polarity and subjectivity of sentiment scores [10].The TextBlob returns two scores: one indicating the sentiment polarity and the other indicating the sentiment subjectivity. The sentiment polarity score ranges from -1–1, with higher scores indicating a more positive sentiment. The sentiment subjectivity score ranges from 0 to 1, with higher scores indicating a more subjective sentiment. This study calculated the sentiment score by creating a TextBlob object initialized using a nursing record string and extracting the ‘ sentiment ‘ attribute from the object. Each individual nursing note of the patient was calculated to obtain an emotional polarity score and an emotional subjectivity score. Then the average score of each patient’s polarity and subjectivity was calculated as the patient’s two nursing emotional characteristic indicators.

### 2.4 Indicators

In this study, the primary outcome was one-year mortality.

Indicators with more than 30 missing data were excluded. Indicators included age, glucose, sentiment polarity, sentiment subjectivity, systolic blood pressure (SBP), diastolic blood pressure (DBP), platelets, anion gap, chloride, creatinine, red cell distribution width (RDW), white cell count (WBC), red blood cell count (RBC), Charlson Comorbidity Index (CCI), Glasgow coma scale (GCS), Sequential Organ Failure Assessment (SOFA), Simplified Acute Physiology Score II (SAPS II), Oxford acute severity of illness score (OASIS), systemic inflammatory response syndrome (SIRS), LOS (Length of stay), Length of stay in ICU, gender (male, female), tobacco (yes, no), alcohol (yes, no), surgery (yes, no), diabetes (yes, no), hypertension (yes, no), renal disease (yes, no), coronary artery disease (yes, no), mechanical ventilation (yes, no), sepsis shock (yes, no), acute kidney injury (AKI) stage (0,1,2,3).

One-year mortality: the patient dies within one year after discharge from the hospital due to illness or other reasons.

### 2.5 Statistical analysis

R language was used for data analysis. The median (P_25_-P_75_) was used to describe the quantitative data of non-normal distribution, and the Mann-Whitney U test was used to compare between groups. Qualitative data were described by composition comparison, and the chi-square test was used to compare between groups. The box plot was used to compare the differences in sentiment polarity/sentiment subjectivity between different groups. Multivariate logistic regression was used to explore the independent correlation between sentiment polarity/sentiment subjectivity and one-year mortality of elderly sepsis patients after controlling for confounding variables, with a significant difference between the two groups. Ridge regression, XGBoost regression, and random forest were used to explore the importance of sentiment polarity and subjectivity in the one-year mortality of elderly sepsis patients. Restricted cubic spline (RCS) was used to explore whether there was a linear relationship between sentiment polarity, sentiment subjectivity and the one-year mortality of elderly sepsis patients. Kaplan-Meier (KM) curve was used to explore the relationship between the sentiment polarity (or sentiment subjectivity) and the 1-year death of the patient. P < 0.05 is considered to be statistically significant.

## 3 Results

### 3.1 Patient baseline information

A total of 5305 patients with sepsis were enrolled in this study, of which 3632 patients died within one year. As can be seen from **Table 1**, there were differences in age, sentiment polarity, sentiment subjectivity, RDW, WBC, RBC, anion gap, chloride, CCI, GCS, SAPS II, OASIS, LOS, Length of stay in ICU, tobacco, alcohol, coronary artery disease, diabetes, hypertension, separate shock and AKI stage.

**Table 1:** Patient baseline data.

Variable		Control group	Dead group	P
Age		74.000 [67.000,81.000]	77.000 [69.000,84.000]	<0.001
Glucose		105.000 [89.000,128.000]	107.000 [89.000,130.000]	0.283
Sentiment polarity		0.045 [0.025,0.065]	0.042 [0.022,0.060]	<0.001
Sentiment subjectivity		0.357 [0.327,0.386]	0.373 [0.346,0.399]	<0.001
SBP		89.000 [79.000,98.000]	88.000 [79.000,98.000]	0.985
DBP		42.000 [36.000,48.000]	42.500 [37.000,49.000]	0.083
RDW		14.500 [13.600,15.800]	14.900 [13.800,16.400]	<0.001
WBC		9.100 [6.400,12.700]	9.800 [6.800,13.600]	<0.001
RBC		2.900 [2.510,3.350]	2.780 [2.410,3.260]	<0.001
Platelets		169.000 [120.000,234.000]	178.000 [118.000,246.000]	0.053
Anion gap		13.000[11.000,15.000]	13.000 [11.000,15.000]	<0.001
Chloride		102.000 [98.000,106.000]	102.000 [97.000,106.000]	0.004
Creatinine		1.100 [0.800,1.700]	1.100 [0.800,1.700]	0.139
CCI		6.000 [5.000,8.000]	7.000 [6.000,9.000]	<0.001
GCS		15.000 [15.000,15.000]	15.000 [14.000,15.000]	<0.001
SOFA		3.000 [2.000,4.000]	3.000 [2.000,4.000]	0.179
SAPS II		40.000 [33.000,48.000]	43.000 [37.000,51.000]	<0.001
OASIS		33.000 [28.000,39.000]	35.000 [29.000,41.000]	<0.001
SIRS		3.000 [2.000,3.000]	3.000 [2.000,3.000]	0.119
LOS		8.107 [5.195,13.818]	10.113 [6.047,17.740]	<0.001
Length of stay in ICU		2.436 [1.393,4.830]	3.012 [1.728,5.999]	<0.001
Gender	male	933 (55.768)	1950 (53.689)	0.158
	female	740 (44.232)	1682 (46.311)	
Surgery	no	236 (14.106)	526 (14.482)	0.717
	yes	1437 (85.894)	3106 (85.518)	
Tobacco	no	1317 (78.721)	3010 (82.874)	<0.001
	yes	356 (21.279)	622 (17.126)	
Alcohol	no	1463 (87.448)	3333 (91.768)	<0.001
	yes	210 (12.552)	299 (8.232)	
Rental disease	no	1064 (63.598)	2393 (65.887)	0.104
	yes	609 (36.402)	1239 (34.113)	
Coronary artery disease	no	1106 (66.109)	2569 (70.732)	<0.001
	yes	567 (33.891)	1063 (29.268)	
Diabetes	no	976 (58.338)	2269 (62.472)	0.004
	yes	697 (41.662)	1363 (37.528)	
Hypertension	no	981 (58.637)	2237 (61.591)	0.041
	yes	692 (41.363)	1395 (38.409)	
Mechanical ventilation	no	1484 (88.703)	3239 (89.180)	0.606
	yes	189 (11.297)	393 (10.820)	
Sepsis shock	no	1442 (86.192)	2941 (80.975)	<0.001
	yes	231 (13.808)	691 (19.025)	
AKI stage	0	462 (27.714)	953 (26.341)	<0.001
	1	274 (16.437)	513 (14.179)	
	2	594 (35.633)	1228 (33.941)	
	3	337 (20.216)	924 (25.539)	

SBP: systolic blood pressure, DBP: diastolic blood pressure, RDW: red cell distribution width, WBC: white cell count, RBC: red blood cell count, CCI: Charlson Comorbidity Index, GCS: Glasgow coma scale, SOFA: Sequential Organ Failure Assessment, SAPS II: Simplified Acute Physiology Score II, OASIS: Oxford acute severity of illness score, SIRS: systemic inflammatory response syndrome, Length of stay: LOS, ICU: Intensive Care Unit, AKI: acute kidney injury

**Fig 1:**
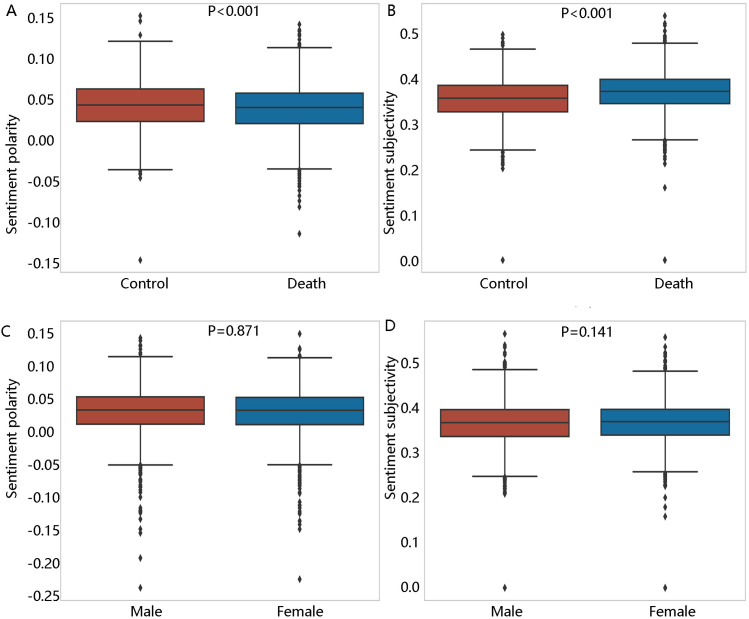
The box plot of sentiment polarity/sentiment subjectivity between different groups, (A) sentiment polarity between control and death group, (B) sentiment subjectivity between control and death group, (C) sentiment polarity between male and female group, (D) sentiment subjectivity between male and female group.

As shown in Fig 1A-1B, compared with the control group, the one-year mortality group year had lower sentiment polarity and higher sentiment subjectivity. However, there was no difference in sentiment polarity between men and women (P = 0.871, **Fig 1C**). The same result was found in sentiment subjectivity (P = 0.141, **Fig 1D**).

### 3.2 Correlation between sentiment polarity/sentiment subjectivity and one-year mortality of elderly sepsis patients

We used multivariate logistic regression to further explore whether sentiment polarity and sentiment subjectivity were independently related to the one-year mortality of elderly sepsis patients. The results of multivariate logistic regression (**Table 2**) showed that sentiment polarity and sentiment subjectivity were independently related to one-year mortality of elderly sepsis patients after adjusting for confounding variables, in which sentiment polarity was a protective factor and sentiment subjectivity was a risk factor.

**Table 2:** Correlation between sentiment polarity/sentiment subjectivity and one-year mortality of elderly sepsis patients.

Predictor	OR (95%CI)	P
Intercept	0.249 (0.06,1.038)	0.056
Sentiment polarity	0.012 (0.001,0.109)	<0.001
Sentiment subjectivity	37.058 (13.43,102.075)	<0.001
Age	1.020 (1.013,1.028)	<0.001
CCI	1.187 (1.152,1.223)	<0.001
OASIS	1.018 (1.008,1.028)	<0.001
SAPS II	0.995 (0.988,1.002)	0.186
GCS	0.925 (0.895,0.956)	<0.001
RDW	1.049 (1.025,1.074)	<0.001
WBC	1.011 (1.002,1.020)	0.017
RBC	0.865 (0.777,0.963)	0.008
Anion gap	0.997 (0.977,1.017)	0.737
Chloride	0.988 (0.979,0.997)	0.009
LOS	1.021 (1.013,1.030)	<0.001
Length of stay in ICU	1.010 (0.996,1.024)	0.176
Tobacco	0.902 (0.771,1.057)	0.200
Alcohol	0.656 (0.534,0.806)	<0.001
AKI stage 1	0.840 (0.690,1.023)	0.082
AKI stage 2	0.843 (0.713,0.995)	0.044
AKI stage 3	0.876 (0.714,1.076)	0.207
Sepsis shock	1.165 (0.980,1.388)	0.085
Coronary artery disease	0.845 (0.738,0.967)	0.014
Diabetes	0.704 (0.615,0.805)	<0.001
Hypertension	0.904 (0.795,1.028)	0.122

RDW: red cell distribution width, WBC: white cell count, RBC: red blood cell count, CCI: Charlson Comorbidity Index, GCS: Glasgow coma scale, SAPS II: Simplified Acute Physiology Score II, OASIS: Oxford acute severity of illness score, Length of stay: LOS, ICU: Intensive Care Unit, AKI: acute kidney injury

Three machine learning models explored the independent factors of one-year mortality of elderly sepsis patients, and the results (**Table 3**) suggested that sentiment polarity and sentiment subjectivity were major factors.

**Table 3:** The importance of sentiment polarity and subjectivity in the one-year mortality of elderly sepsis patients.

Ridge regression		XGBoost regression		Random forest	
Variable	Weight Importance	Variable	Weight Importance	Variable	Weight Importance
Sentiment polarity	0.992	Sentiment subjectivity	533	Sentiment subjectivity	0.122
Sentiment subjectivity	0.761	LOS	543	LOS	0.114
Alcohol	0.089	Sentiment polarity	635	Sentiment polarity	0.106
Diabetes	0.068	RBC	504	RBC	0.096
Coronary artery disease	0.038	RDW	427	WBC	0.095
CCI	0.031	WBC	471	RDW	0.091
RBC	0.031	Age	542	OASIS	0.082
GCS	0.013	OASIS	389	Age	0.078
RDW	0.01	CCI	278	Chloride	0.074
AKI stage	0.008	Chloride	338	CCI	0.059

RDW: red cell distribution width, WBC: white cell count, RBC: red blood cell count, CCI: Charlson Comorbidity Index, GCS: Glasgow coma scale, OASIS: Oxford acute severity of illness score, Length of stay: LOS, AKI: acute kidney injury

**Fig 2:**
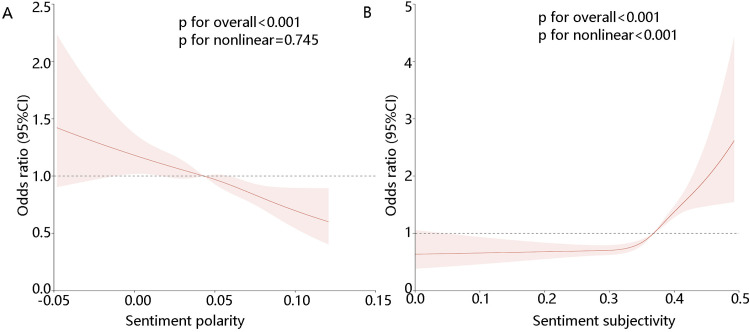
The linear relationship between sentiment polarity, sentiment subjectivity and the one-year mortality of elderly sepsis patients, (A) sentiment polarity, (B) sentiment subjectivity.

**Fig 3:**
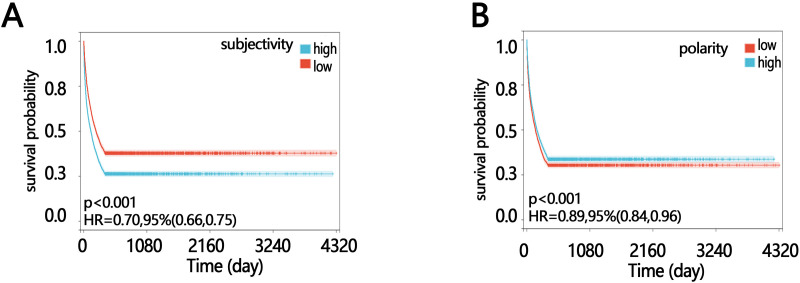
The KM of sentiment polarity and sentiment subjectivity, (A) sentiment subjectivity, (B) sentiment polarity.

We further explored whether there was a linear relationship between sentiment polarity, sentiment subjectivity and the one-year mortality of elderly sepsis patients. **Fig 2A-2B** showed that there was a linear relationship between sentiment polarity and the one-year mortality of elderly sepsis patients. At the same time, there was a nonlinear relationship between sentiment subjectivity and the one-year mortality of elderly sepsis patients. KM results showed that high subjectivity was beneficial to the 1-year survival of patients (Fig 3A, HR = 0.70 95% CI [0.66,0.75] p < 0.05), and low polarity was advantageous to the 1-year survival of patients (Fig 3B, HR = 0.89,95% CI [0.84, 0.96])(The high and low groups were divided according to the median.).

## 4 Discussion

### 4.1 Nursing sentiment and prognosis

This study was based on the MIMIC-IV database to explore the correlation between the sentiment of nursing notes and the one-year mortality of sepsis patients. We found that sentiment polarity and sentiment subjectivity were independently related to one-year mortality of elderly sepsis patients.

Emotional scores in nursing notes reflect the health care provider’s attitude toward the patient. This emotion or attitude generally arises when the health care provider communicates with the patient. Therefore, the emotional attitude of the health care provider not only reflects the physician’s assessment of the patient’s current physical status, but is also based to some extent on feedback about the efficacy of the patient’s treatment. This feedback largely influences the patient’s adherence to subsequent treatment [11,12]. Specifically, the positive mood of the healthcare provider towards the patient indicates that the patient’s treatment is working well. After receiving such feedback, the patient’s trust in the treatment is increased, which makes them more willing to follow the medical advice and cooperate with the treatment. A more positive attitude and compliance with treatment lead to better patient outcomes. This results in a positive cycle that accelerates patient healing. For example, a cohort study of diabetic patients showed that a highly empathetic physician’s positive attitude toward the patient significantly increased the patient’s adherence to treatment, which in turn increased the rate of glycohemoglobin control [13]. In addition, healthcare professionals’ attitudes affect patients’ psychological state and physiological responses. Positive emotions reduce patients’ anxiety and depression levels. A randomized controlled trial showed that nurses trained in communication built better trust with patients, which led to lower postoperative anxiety and pain [14]. Chronic stress due to illness activates the hypothalamic-pituitary-adrenal (HPA) axis, leading to elevated cortisol and suppressed immune function [15]. Physicians’ emotions toward patients may affect the patients’ HPA axis, influencing their levels of inflammatory markers and thus their prognosis [16,17].

In this study, we found that sentiment polarity and sentiment subjectivity were independently related to one-year mortality of elderly sepsis patients. Sentiment polarity was negatively related to one-year mortality of elderly sepsis patients and sentiment subjectivity was positively related to one-year mortality of elderly sepsis patients. A study based on the MIMIC-III database found that sentiment polarity and sentiment subjectivity were independently associated with the 28-day hospitalization mortality of sepsis patients, and both sentiment polarity and sentiment subjectivity were negatively related to the 28-day hospitalization mortality of sepsis patients [18]. A similar relationship was also found in another study that explored the emotion of nursing notes and the 30-day death of sepsis patients [19]. Our results were inconsistent with those of other people’s research, which may be mainly related to the inconsistency of the selected research objects and research nodes. Specifically, the dependent variables in Gao et al. ‘s [18] and Zhou et al. ‘s [19] studies were 28-day and 30-day in-hospital deaths, respectively, whereas the dependent variable in our study was 1-year mortality. Different criteria for exclusion, which in turn lead to heterogeneity in the study population. Their studies included 1851 and 1844 patients, respectively, whereas our study included 5305 patients. The sentiment of medical staff’s notes not only affected the prognosis of sepsis patients but also affected the prognosis of other diseases. McCoy and others conducted sentiment analysis on the discharge records of psychiatric inpatients and patients in general medical units from 2011 to 2014, and found that more positive sentiments were related to patients’ lower risk of readmission [20]. In another study of severe acute renal failure, it was found that compared with the survival group, the sentiment subjectivity score of the death group was higher, the sentiment polarity score was lower, and the prediction model with sentiment score had better prediction performance [21]. Another study found similar results [8]. Studies on the correlation between sentiment polarity and prognosis have found a negative correlation between emotional polarity and prognosis, indicating that the more positive the sentiment of medical staff, the better the prognosis of patients. This also suggested that medical staff need to maintain a positive attitude in the daily diagnosis and treatment process.

### 4.2 Other factors and prognosis in sepsis

In this study, we also found some influencing factors of one-year mortality of elderly sepsis patients, such as age, CCI, and RDW. Many studies have found that age is an influential factor in the death of sepsis patients. Compared with young people, elderly patients have weakened physical resistance and impaired physiological barriers. CCI is the most commonly used comorbidity assessment tool at present. The higher the score, the more serious the number and degree of underlying diseases are. Elderly patients often have more basic diseases (comorbidities), and many common comorbidities increase the risk of infection and death [22]. RDW is a parameter reflecting the volume heterogeneity of peripheral red blood cells, which is closely related to the prognosis of many diseases. At the present stage, most studies focus on the influence of patients themselves on the prognosis, and few studies pay attention to the influence of medical staff. This study suggests that we should also pay attention to the influence of medical staff’s behavior or sentiment on the prognosis of diseases in subsequent studies.

### 4.3 Strengths and limitations

This study explored the relationship between the emotional score of nursing notes and the death of elderly patients with sepsis within one year. We found its potential in predicting the death of patients within one year, which suggested the application value of nursing notes in clinical practice. However, some limitations also existed in this study. Firstly, due to the limited data in the database, this study lacked some information about biomarkers of sepsis patients, such as Uncoupling Protein 2. Secondly, this study only revealed the association and cannot determine the causal relationship between them. Thirdly, TextBlob has a weak ability to analyze complex sentences, and may have errors in the emotional direction of complex sentences in nursing notes and form bias for subjectivity and polarity scores, which affects the accuracy of the conclusion. Fourthly, the subjective nature of nursing notes may be influenced by the personality of the health care provider. Fifthly, the study was not validated with external data due to the lack of suitable external data at present. In the future, we will conduct data collection in our hospital to validate the findings. Sixthly, this study was a single-center retrospective study, which inevitably had some bias. For example, MIMIC comprises deidentified health-related data from patients who were admitted to the critical care units of the Beth Israel Deaconess Medical Center. In the United States, this center is a top 3 hospital with a suitable staff ratio and a high level of nurse experience. Therefore, the conclusion of this study may only be suitable for such large hospitals. For other small hospitals, the conclusion may not be suitable. In addition, patients from a single center are biased and heterogeneous with patients from other hospitals (such as disease severity, race, social status, family economics, education level, etc.). These results may result in the conclusions obtained in this study not being able to be verified in patients from other hospitals.

### 4.4 Future research directions

Future research should focus on exploring the causal relationship between sentiment scores in nursing notes and patient prognosis via well-designed clinical trials, detailing the mechanisms between the two. Then, develop a multimodal prediction model that integrates sentiment analysis of nursing texts with traditional clinical scoring systems, and enhance the generalization of the model through multicenter validation; at the same time, it is necessary to build a real-time automated monitoring tool, incorporate dynamic sentiment indicators into the clinical decision support system to achieve early warning of high-risk patients, and deeply analyze the intrinsic relationship between sentiment characteristics (such as negative/positive word combination patterns) and pathophysiological mechanisms. In addition, the impact of cross-language and cultural differences on the validity of sentiment analysis needs to be addressed urgently, and adaptive algorithms (such as Chinese subjective dictionary construction technology) need to be developed to improve the universality of application in different medical contexts.

## 5 Conclusions

The sentiment of nursing notes was correlated with the one-year mortality of elderly sepsis patients.

## Supporting information

S1 File Raw data. (XLSX)
